# Surgery

**Published:** 1897-09

**Authors:** W. H. Harsant


### SURGERY.

**Painful affections of the sole of the foot without obvious lesion** have been the subject of study by surgeons in all countries for many years past, and numerous cases of neuralgic affections of the sole of the foot have been recorded under the generic title of "Neuralgia," or the more particular title of "Metatarsalgia"; but the first who made a scientific study of the subject was Thomas G. Morton, of Philadelphia, who published his classical paper, entitled "A Peculiar and Painful Affection of the Fourth

1 *Lancet*, 1896, ii. 173. 2 *Loc. cit.* 3 *Loc. cit.*
4 *Am. J. M. Sc.*, 1897, cxiii. 511.

Metatarso-Phalangeal Articulation," in 1876.[1] Since that time the affection has been universally spoken of by the name of "Morton's Painful Foot," and it seems right that this name should be perpetuated, inasmuch as Dr. Morton was the first to demonstrate the true pathology as well as the treatment of this hitherto obscure and intractable affection. He pointed out that the pain was always localised in the fourth metatarso-phalangeal articulation; in some instances it followed at once after an injury of the foot, in others it was gradually developed from pressure, while in others there was no recognised cause. Of fifteen cases recorded by him, thirteen were in females and only two were in males.

**A typical case** was as follows: A lady, while walking down a steep ravine in Switzerland, trod upon a large stone which rolled from under her foot, causing her to slip, throwing her entire weight upon the forward foot; though not falling, she found the right foot injured; the pain was intense and accompanied by fainting sensations. After this she found it impossible to wear a shoe even for a few moments, the least pressure inducing an attack of severe pain. At no time did the foot or toe swell or present any evidence of having been injured. During the succeeding five years the foot was never entirely free from pain; often her suffering was very severe, coming on in paroxysms. She could wear no shoe except a very large one, and that only for a limited space of time, invariably being obliged to remove it every half-hour or so to relieve the foot. The pain was always referred to the metatarso-phalangeal joint of the fourth toe; during the severe paroxysms it extended, occasionally, to the knee. There was neither redness nor swelling anywhere about the foot. The head of the fourth metatarsal, with the phalangeal base, and the soft parts about the joint, were exceedingly sensitive.

This case was a type of all the cases met with by Dr. Morton, and the explanation of the cause of the affection as given by him is as follows: The metatarso-phalangeal joints of the first, second, and third toes are often found on a line with each other; the head of the fourth metatarsal is found to be from one-eighth to one-fourth of an inch behind the head of the third; while the head of the fifth is from three-eighths to half-an-inch behind the head of the fourth. Thus, while the joint of the third is slightly above, the joint of the fifth is considerably below the metatarso-phalangeal articulation of the fourth; the base of the first phalanx of the little toe is thus brought on a line with the head and neck of the fourth metatarsal, and the head of the fifth opposite the neck of the fourth. It will further be found that lateral pressure brings the head of the fifth metatarsal and the little toe into direct contact with the base of the first phalanx, and head and neck of the fourth, and to some extent the extremity of the fifth metatarsal rolls above and under this bone.

[1] *Am. J. M. Sc.*, 1876, lxxi. 37.

The superficial branch of the external plantar nerve separates into two digital branches which supply the outer and inner sides of the fifth toe, and the outer side of the fourth; small branches being distributed between the fourth and fifth toes, about the metatarso-phalangeal joints. These nerves are liable to be bruised or pinched between the fourth and fifth metatarsals, where movement is freer than between the others, and this accounts for the neuralgia occurring here. Dr. Morton's explanation, although not altogether satisfactory, is probably the best that can be offered, and no better one has since been advanced. The treatment adopted and recommended by him in all severe cases was excision of the fourth metatarso-phalangeal articulation, which in every case had the desired effect of completely curing the pain, without materially interfering with the usefulness of the foot.

Many other writers afterwards recorded cases of Morton's Painful Foot, and all of them continued to recommend the **treatment by excision of the joint,** particularly Dr. Thomas S. K. Morton, of Philadelphia, in 1893,[1] who, I suppose, was a son or relative of the Thomas G. Morton who gave his name to the affection. Other writers included Dr. Auguste Pollosson[2] of Lyons, L. G. Guthrie,[3] and E. H. Bradford.[4]

In 1893 an article appeared by Dr. N. F. Graham, Professor of Surgery in the Medical Department of Howard University, Washington, entitled "Neuralgia of the Base of the Fourth Toe, and its Treatment,"[5] in which was first advocated a method of **treatment by excision of a portion of the branch of the external plantar nerve** supplying the adjacent sides of the fourth and fifth toes. He records four cases of this operation, in all of which the resection of the nerve proved so satisfactory that he says he shall continue to practise this mode of treatment in preference to any other. It has the advantage of being simple and easily performed. It leaves no deformity, and seems to be a rational mode of procedure. If, as seems to be established from the cases cited, the pain is due to the nerve, then this plan for its cure is reasonable and worthy of trial in these cases, which are by no means rare. The removal of the toe and part of its metatarsal bone, or a resection by Morton's method, leaves some degree of deformity, which is an objection, while removal of a portion of the nerve leaves a mere scar on the sole of the foot without any deformity or weakness whatever.

It is curious to me that writers on the subject of this affection during the last three or four years should have paid so little attention to **Dr. Graham's method of treatment.** I attribute much of this neglect to the unfortunate title of the paper, which has caused it to be indexed as "Neuralgia" and not, as it should be, as "Morton's Painful Foot" or "Metatarsalgia." It was only so recently as last summer that a paper was read on this

1 *Ann. Surg.*, 1893, xvii. 680. 2 *Lancet*, 1889, i. 436. 3 *Ibid.*, 1892, i. 628.
4 *Boston M. & S. J.*, 1891, cxxv. 52. 5 *Med. News*, 1893, lxii. 512.

subject by Mr. A. H. Tubby at the annual meeting of the British Medical Association in Carlisle, where he makes no reference whatever to the treatment by excision of nerve, but recommends palliative treatment of various kinds, and in severe cases excision of the metatarso-phalangeal joint.

I was myself a sufferer from this painful affection of the foot for five years, from 1889 to 1895, and I may be pardoned for alluding to my own case, which was almost exactly similar to the case of Dr. Morton's described above and to most of the cases met with by others. It was suddenly produced while playing at lawn tennis in a thin-soled canvas shoe. I felt a sudden tearing and cutting pain in the sole of the foot immediately under the fourth metatarso-phalangeal joint. The pain was so severe that I was unable to walk on the foot for several days, and even after that I could only walk with much pain. I did not realise the true condition of affairs, but thought that I had ruptured the transverse metatarsal ligament, and finding that the pain and inability to walk continued, I put my foot in a plaster-of-Paris splint and kept it at rest for a month; but on taking off the splint at the end of that time the pain on walking was as bad as ever. I consulted many of my surgical friends, and eventually I read Dr. Morton's paper, which at once shewed me the true condition of affairs. I was reluctant to have amputation of the toe or excision of the joint performed; but was making up my mind to the latter course when Dr. Graham's paper attracted my attention, and I at once decided to have the nerve excised. I persuaded my lamented friend, the late Mr. J. Greig Smith, to perform this operation for me, which he did most skilfully in 1894, having first practised the operation in the dead-house on three or four occasions with my assistance. The result of the operation was a perfect and complete cure, and I have no hesitation in recommending this operation in preference to all others.

Palliative treatment in severe cases is useless. I found the greatest relief to be obtained from blocking up the sole of the boot with a piece of leather just behind the tender spot, so as to take the weight off the painful part. All the other devices mentioned by Mr. Tubby were perfectly useless in my case, and there was a complete absence of the corn on the sole of the foot of which he speaks and of which no mention is made by Morton or other writers. Indeed, I cannot help thinking that Mr. Tubby has not met with a true case of Morton's Painful Foot, but has confused a case of painful corn on the sole of the foot with Morton's disease.

W. H. Harsant.

